# Identification of novel mutations and functional impacts of EPAS1 in colorectal cancer

**DOI:** 10.1002/cam4.4116

**Published:** 2021-07-11

**Authors:** Farhadul Islam, Vinod Gopalan, Cu Tai Lu, Suja Pillai, Alfred K. Lam

**Affiliations:** ^1^ School of Biomedical Sciences Faculty of Medicine University of Queensland Brisbane Queensland Australia; ^2^ School of Medicine and Dentistry Griffith University Gold Coast Queensland Australia; ^3^ Department of Biochemistry and Molecular Biology University of Rajshahi Rajshahi Bangladesh; ^4^ Department of Surgery Gold Coast University Hospital Southport Queensland Australia

**Keywords:** cancer genetics, cancer prognosis, cell proliferation, colorectal cancer, EPAS1, invasion

## Abstract

Endothelial PAS domain‐containing protein 1 (EPAS1) has implications in many cancers. However, the molecular behaviours, functional roles and mutational status of *EPAS1* have never been studied in colorectal carcinoma (CRC). The study aims to examine the genetic alterations and functional roles of *EPAS1* in CRC. In addition, the clinical impacts of *EPAS1* in CRC were studied. Significant *EPAS1* DNA amplification (63.4%; *n *= 52/82) and consequent mRNA overexpression (72%; *n* = 59/82) were noted in patients with CRC. In CRC, 16% (*n* = 13/82) of the patients had mutations in the *EPAS1* coding sequence and most of the mutated samples exhibited aberrant DNA changes and mRNA overexpression. We have identified two novel variants, c.1084C>T; p.L362L and c.1121T>G; p.F374C in CRC. These *EPAS1* aberrations in CRC were correlated with clinicopathological parameters, including tumour size, histological grade, T‐stages, cancer perforation as well as the presence of synchronous cancer. Also, reduced cell proliferation, wound healing, migration and invasion were noted in colon cancer cells followed by *EPAS1* silencing. To conclude, the results obtained from the current study indicated that *EPAS1* plays important role in colorectal carcinogenesis, thus, could be useful as a prognostic marker and as a target for therapy development.

## Graphical abstract

The novel findings of EPAS1 mutation in cancer tissues from colon cancer might be involved in carcinogenesis. Thus, the molecular and functional studies implied that EPAS1 plays crucial roles in CRC pathogenesis and have the potential to be used as a prognostic marker and as a therapeutic target.

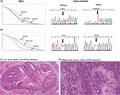

## 1 INTRODUCTION

In low oxygen tension (hypoxia), the hypoxia‐inducible factor 1 (HIF1) interacts with the hypoxia response element, resulting in the regulation of many genes expression involved with iron, glucose metabolism, cell proliferation, angiogenesis and cells’ survival.^1^, ^2^ HIF1β subunit is constitutively expressed, whereas the HIF1α subunit undergoes post‐translational degradation under normal oxygen concentration condition.^3^ The functioning HIF1 is a heterodimer, composed of a β and an α or 2α and HIF3α subunits. These HIF1α isomers are encoded by the *HIF1A*, *EPAS1* and *HIF3A* genes, respectively.^3^ The HIF2α also known as EPAS1 (endothelial PAS domain‐containing protein 1 is an oxygen‐sensitive component of HIF1).

Several cancers including colorectal, glioma, pheochromocytoma, neuroblastoma, hepatic carcinoma, renal carcinoma and non‐small cell lung carcinomas are associated with the molecular deregulation of *EPAS1*.^4^, ^5^, ^6^, ^7^, ^8^, ^9^, ^10^, ^11^, ^12^, ^13^ In colorectal carcinoma (CRC), the expression of EPAS1 has been associated with the pathological stages, histological grade, size, recurrence and survival of the patients.^7^, ^13^, ^14^, ^15^, ^16^, ^17^, ^18^, ^19^ For example, the expression of EPAS1 protein is inversely associated with the higher grade of tumours.^17^ *EPAS1* mRNA level in plasma was correlated with advanced stages (stage III and stage IV) and poor survival of patients with CRC.^18^ In addition, the expression of EPAS1 protein was directly associated with micro‐vessel density and cyclooxygenase two expressions (the mediator of angiogenesis) in tissue samples of patients with CRC.^14^ In contrast, Imamura and colleagues noted that the loss of EPAS1 expression was strongly associated with advanced tumour stage along with increased vascular endothelial growth factor (VEGF) expression and increased in vitro angiogenesis.^15^ Accordingly, suppression of EPAS1 induced enhanced anchorage‐independent tumour growth in vitro and tumour formation in vivo in xenotransplant mouse.^15^ Furthermore, a significantly reduced expression of *EPAS1* mRNA was observed in primary CRC tissues when compared to non‐cancerous colorectal tissues.^13^ Nevertheless, the molecular roles of EPAS1 in the pathogenesis of CRC remain controversial.

**FIGURE 1:**
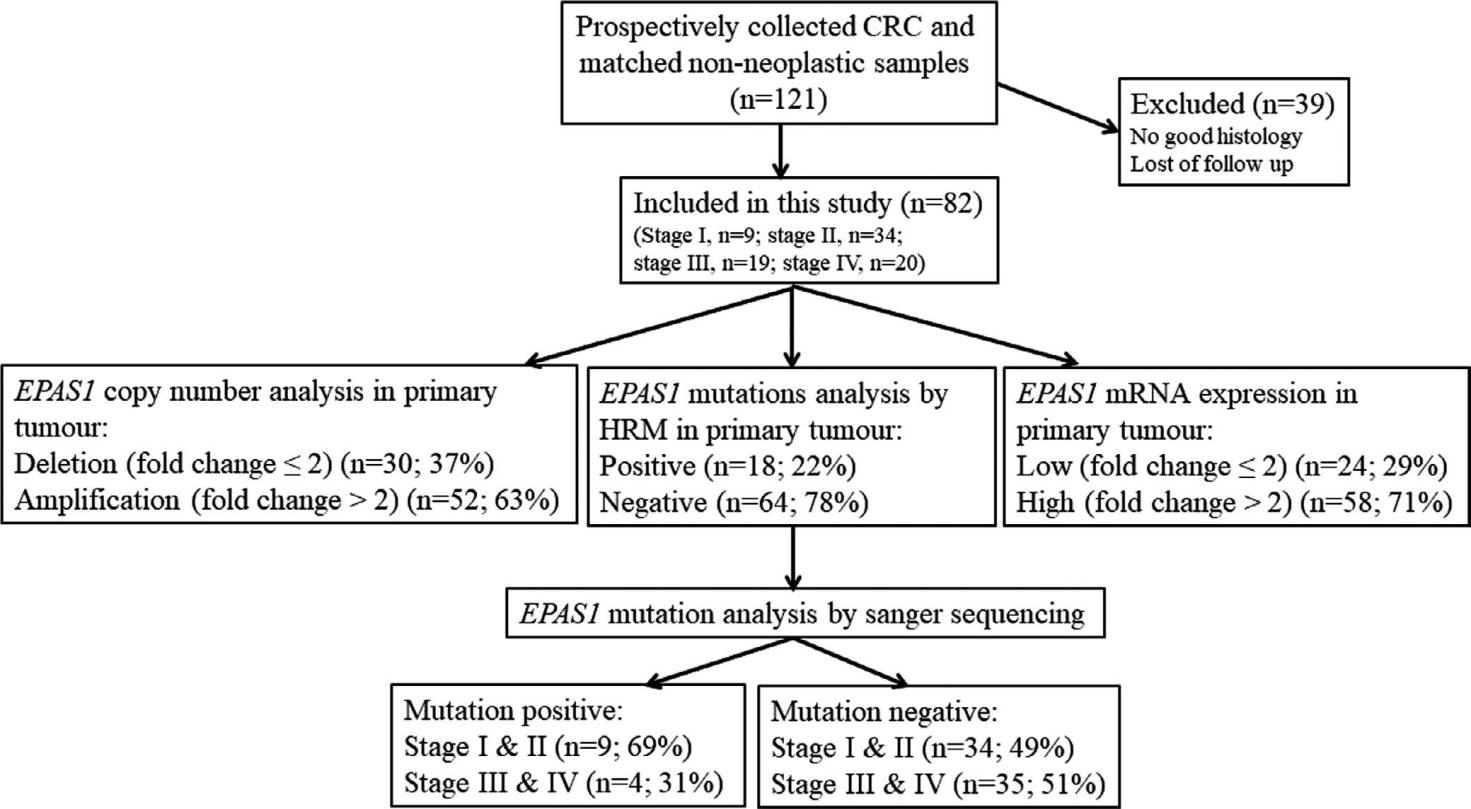
Schematic flow diagram of the methodology used for clinical samples analysis in the current study. Patients with colorectal carcinoma (CRC) (stages I to IV) who had surgical resection of tumours by a specific surgeon were included in this study. Whereas patients with no follow‐up and poor histology were excluded from the present study

Mutations in the *EPAS1* sequence are associated with several neoplasms, including paraganglioma, pheochromocytoma and pancreatic adenocarcinoma.^10^, ^20^, ^21^, ^22^ Lynch syndrome, also known as hereditary non‐polyposis colorectal cancer, is a type of familial cancer syndrome. Patients and their family members have an increased risk of development of the different types of cancers, especially CRC.^23^ Mutations in *EPAS1* are also noted in patients with Lynch syndrome. However, the screening of *EPAS1* mutations, molecular deregulations of *EPAS1* and their Clinicopathological implications in CRC patients has not been described in the English literature. Thus, we aim to screen *EPAS1* mutations in CRC tissue samples and to examine the association of the mutations with different clinicopathological factors. In addition, *EPAS1* DNA copy number and mRNA expression changes and their clinicopathological correlations as well as *EPAS1* induced cellular changes in colon cancer cells were investigated. The flow diagram of the experimental design of the current study is given in Figure 1.

## 2 MATERIALS AND METHODS

### 2.1 Patients and Clinicopathological features

Tumour and matched non‐cancer (near the surgical resection margin) tissues from the patient who underwent resection of CRCs were prospectively collected from 2012 to 2014. The samples were collected consecutively during the period of study with no selection bias and the collected samples were stored at −80℃ followed by snap‐frozen in liquid nitrogen. This study only included conventional adenocarcinoma. Tissue samples with not enough tumour for histological examination and with no patient’s follow‐up information were not included in this study. Ethics approval was granted for the study and written informed approval was acquired from all the adult individuals (over 18 years) for the use of clinical data in research. All the methods were carried out in accordance with relevant guidelines and regulations.

Tumour tissues were sectioned, stained with haematoxylin & eosin for light microscopic histological examination. Clinicopathological parameters of the samples were analysed and recorded from the histological examination. Then, they were graded, classified and staged pathologically following the Fifth edition of the World Health Organization (WHO) classification of Digestive system tumours guidelines.^24^ The demographical data, presence of tumour perforation, associated adenoma and synchronous carcinomas of the patients were recorded. Tumour perforation is perforation at the site of tumour and could increase the likelihood of tumour spread. Adenoma is a precursor of adenocarcinoma. In this study, the presence of adenoma was recorded at the time of pathological examination, before or follow‐up after the surgery of adenocarcinoma. Synchronous CRC is defined as the presence of more than one primary CRC noted at the time of surgery.^25^ The presence of synchronous cancer increases the tumour volume and has an impact of patients’ management.

After reviewing the histology, 82 patients (female 42; male 40) with CRC were included for analysis in this study. The age range of the patients was 31–91 years with a mean age of 68 years. The site of the cancer in the colorectum and size (mm) of the tumour were documented followed by the macroscopic examination of the resected specimen. Carcinomas in the caecum, ascending and transverse colon is defined as proximal carcinomas (right sided cancers), whereas cancers in descending, sigmoid colon and rectum were categorized as distal carcinomas (left sided cancers). Overall, 83% (*n* = 68) of the cancers were proximal carcinomas, while 17% (*n* = 14) were distal carcinomas. Also, 21% (*n* = 17) of the carcinomas had lymph node metastasis. There were 11% (*n *= 9) stage I, 41.5% (*n* = 34) stage II, 23.1% (*n* = 19) stage III and 24.4% (*n *= 20) stage IV carcinomas.

A multi‐disciplinary pre‐agreed standardised protocol was used for the clinical management of the patients. Pre‐operative and post‐operative adjuvant therapies were given based on the clinical protocols and pathological parameters of cancer. The interval between the date of surgery and the date of patient’s death or closing date of the study was used as the follow‐up time for the patients with CRC. Whereas the date of surgical resection of the tumours to the last follow‐up or date of death of patients was used to calculate the actuarial survival rate. Also, patient’s death only related to CRC was recorded as the endpoint in the statistical analysis. Furthermore, the persistence or recurrence of CRC was recorded.

### 2.2 Extraction of DNA and RNA

Genomic DNA and total RNA were extracted from cryostat (Leica Biosystems) tissue sections (7 μm). The sections having 70% or more volume of samples as cancer tissue were used for DNA and RNA extractions. DNA was extracted using DNeasy Blood and Tissue kit (Qiagen Pty. Ltd.) from tissues and cells following the manufacturer’s guidelines. Total RNA was extracted using miRNeasy Mini kit (Qiagen) from fresh frozen tissues and cells followed by manufacturer’s guidelines. The purity of the extracted DNA and RNA was checked using a nano‐drop spectrophotometer. The extracted samples are kept at −20℃ for subsequent examinations.

### 2.3 Quantitative real‐time PCR

QuantStudio 6 Flex Real‐Time PCR system (Thermo Fisher Scientific) was used to examine *EPAS1* DNA copy number changes in CRC and adjacent non‐cancerous tissue (*n *= 82) samples. In short, 20 µl of reaction mixture comprising of 10 µl of DyNAmo Flash SYBR green master mix (Bio‐Rad), 1.5 µl of forward/reverse primers (5 µmol/L), 3 µl of DNA (50 ng/µl) and 4 µl of diethylpyrocarbonate‐treated water was used for qPCR according to the previously published protocol.^26^ Couples of genes such as β‐actin, 18s, glyceraldehyde 3‐phosphate dehydrogenase (GAPDH) were sued to normalise the amplification results. Fold changes are used as the level of *EPAS1* DNA number variations. In this study, 2^−[delta][delta]Ct^ equation was used to calculate the fold changes as reported earlier.^27^, ^28^ Fold changes of more than two were used as *EPAS1* DNA amplification, whereas fold changes of two or less were used as *EPAS1* DNA deletion.

For quantitative *EPAS1* mRNA expression analysis, DyNAmo cDNA synthesis kit (Qiagen) was used to generate the cDNA first strand.^27^ Then, a 20 µl of reaction mixture comprising of 10 µl of DyNAmo Flash SYBR green master mix (Bio‐Rad), 1.5 µl of forward/reverse primers (5 µmol/L), 1 µl of cDNA (50 ng/µl) and 4 µl of diethylpyrocarbonate‐treated water was used for qPCR according to the previously published protocol.^26^ *EPAS1* mRNA amplification efficiencies were normalised to multiple genes such as *β*‐*actin*, *18s* and *GAPDH* and the results were shown as the ratio of expression (*EPAS1* mRNA normalised to *GPADH* mRNA). As describe earlier, 2^−[delta][delta]Ct^ equation was used to calculate *EPAS1* mRNA fold changes.^27^ Similar to the DNA copy number changes, fold changes of more than two were used as high *EPAS1* mRNA expression, whereas fold changes of two or less were used as low *EPAS1* mRNA expression. Also, the expression ratio determined by β‐actin showed a similar trend, thus, not presented for redundancy.

### 2.4 High‐resolution melt curve analysis

The presence of mutations in the *EPAS1* sequence (both in carcinomas and matched non‐cancer) was screened by HRM analysis. The target sequence was amplified using the Rotor‐Gene Q detection system (Qiagen). Then, Rotor‐Gene ScreenClust HRM Software was used to analyse HRM curves. A 10 μl of reaction mixture containing 5 μl of 2X SensiMix HRM master mix (Bioline Aust Pty Ltd), 1 μl (30 ng/μl) of DNA, 2 µl of diethylpyrocarbonate‐treated water and 1 μl of forward/reverse *EPAS1* primers (5 µmol/L) was used to PCR amplify the *EPAS1* sequence. The thermal profile used for this analysis was the same as a previously published protocol.^29^ An NTC (no template control) sample was included in each PCR run. Temperature increment from 65 to 85℃ was used to generate the melt curve data with a temperature increase rate of 0.05℃/s and recording fluorescence. The amplicon, primers and product sizes are given in supplementary (SI Table 1).

**TABLE 1:** Relationship between *EPAS1* copy number variations and Clinicopathological factors in colorectal carcinoma (CRC) patients

Features	Number	Amplification	Deletion	*p*‐value
Total patients	82	52 (63.4%)	30 (36.6%)	—
Gender Male	40 (48.8%)	24 (60.0%)	16 (40.0%)	0.34
Female	42 (51.2%)	28 (66.7%)	14 (33.3%)	
Age ≤70	40 (48.8%)	26 (65.0%)	14 (35.0%)	0.47
>70 Site	42 (51.2%)	26 (61.9%)	16 (38.1%)	
Proximal	68 (82.9%)	44 (64.7%)	24 (35.3%)	0.40
Distal	14 (17.1%)	8 (57.1 %)	6 (42.9%)	
Size				
≤40	44 (53.7%)	24 (54.6%)	20 (45.4%)	**0.05**
>40	38 (46.3%)	28 (73.7%)	10 (26.3%)	
Perforation				
Yes	10 (12.2%)	9 (90.0%)	1 (10.0%)	**0.05**
No	72 (87.8%)	43 (59.7%)	29 (40.3%)	
Synchronous cancer				
Yes	7 (8.5%)	7 (100%)	0 (0%)	**0.03**
No	75 (91.5%)	45 (60.0%)	30 (40.0%)	
Grade				
Well	12 (14.6%)	8 (66.7%)	4 (33.3%)	0.96
Moderate	57 (69.5%)	36 (63.2%)	21 (36.8%)	
Poor	13 (15.9%)	8 (61.5%)	5 (38.5%)	
Distant metastasis				
Yes	19 (23.2%)	13 (68.4%)	6 (31.6%)	0.40
No	63 (76.8%)	39 (61.9%)	24 (38.1%)	
Stage				
I	9 (10.9%)	6 (66.7%)	3 (33.3%)	0.86
II	34 (41.5%)	20 (58.9%)	14 (41.1%)	
III	19 (23.2%)	7 (36.9%)	12 (53.1%)	
IV	20 (24.4%)	14 (70.0%)	6 (30.0%)	
Pre‐operative chemo‐radiotherapy				
Yes	6 (7.3%)	3 (50.0%)	3 (50.0%)	0.38
No	76 (92.7%)	49 (64.5%)	27 (35.5%)	

Note

Synchronous cancer means more than one primary cancer in the colon.

### 2.5 PCR products purification and analysis of Sanger sequencing

*EPAS1* variants detected by HRM analysis were validated by Sanger sequencing. NucleoSpin Gel and PCR Clean‐up kit (Macherey‐Nagel) were used to purify the PCR products of mutant samples identified by HRM analysis following the manufacturer’s guidelines. The purified PCR products were sequenced using Big Dye Terminator Chemistry Version 3.1 (Applied Biosystems) under standardised PCR conditions. The 96‐capillary 3730xl DNA analyser (Applied Biosystems) was used to generate the Sanger sequencing and Sequence Scanner 2 software (Applied Biosystems) was used at the Australian Genome Research Facility to analyse the generated data.

### 2.6 Computational prediction

Ensembl transcript ID ENST00000263734 was used as input in computational analysis. Freely available tools such as Mutation Taster (NCBN 37 and Ensembl 69 data release) Protein variation effect analyser (PROVEAN) and Sorting intolerant from tolerant (SIFT) were used to analyse all the variants for their effects on proteins structure and functionality.^30^ Also, ExAc and 1000 Genomes variants databases were checked to identify the novelty of the variants. A −2.5 and 0.05 cut‐off values were used for PROVEAN and SIFT, respectively, for predicting the pathogenicity of the variants in the present study.

### 2.7 Cell culture

SW480, SW48, Lovo and HCT116 colon cancer cell lines and FHC, a non‐neoplastic colon epithelial cell were used in the present study. All the cell lines were purchased from the American type culture collection and the cells were cultured and maintained as described previously.^31^

### 2.8 Transfection of cells

SW480 and Lovo cells were seeded (2 × 10^4^ cells/cm^2^) into 24‐wells plate in Roswell Park Memorial Institute (RPMI)‐1640 medium, supplemented with 10% foetal bovine serum (FBS) and 1% penicillin/streptomycin. After 24h of initial seeding, the cells were transfected using *EPAS1* siRNA silencer (Qiagen) sequence (SW480^−EPAS1^ and Lovo^−EPAS1^) and scramble siRNA (Qiagen) sequence (SW480^Scr^ and Lovo^Scr^) at a concentration of 15 and 10 nM, respectively, according to the previously published protocol with some modifications.^28^ The transfected cells (SW480^−^ *^EPAS1^* and Lovo^−^ *^EPAS1^*), scramble siRNA‐treated cells (SW480^+Scr^ and Lovo^+Scr^) and transfected reagents‐treated cells (SW480^wildtype^ and Lovo^wildtype^) were used for functional assays (SI Figure 1).

### 2.9 Western blot analysis

After 24 h of transfection, the cells were collected and the total proteins were extracted using lysis buffer (Bio‐Rad). Then, the concentration of total proteins was estimated using a BCA assay kit (Thermo Fisher). For blotting, 30 μg of total protein was separated using SDS‐PAGE (4–15% Bio‐Rad mini gels). Then, the Turbo Trans‐blot transfer system was used to transfer the proteins to polyvinylidene fluoride (PVDF) membranes (Bio‐Rad). Subsequently, the membranes were incubated with EPAS1 and GAPDH (mouse monoclonal) antibody at a dilution of 1:1000 overnight with gentle shaking at 4℃. Finally, the membranes were incubated with secondary antibody at a dilution of 1:2000 for 2 h at room temperature and protein signals were detected by following the protocol previously published.^32^

### 2.10 Proliferation assay

The effect of *EPAS1* on colon cancer cell proliferation, a cell counting kit called CCK‐8 (Sigma‐Aldrich) was used as previously described.^33^ Briefly, 1 × 10^4^ SW480 and Lovo cells were seeded in a flat‐bottom 96‐well plate and cultured for 24 h. Then, *EPAS1* siRNA silencer sequence and scramble siRNA sequence were added to the as previously described.^33^ The proliferation of cells followed by transfection on days 0–3 was determined using CCK‐8.

### 2.11 Colony formation assay

The clonogenic capacity of SW480 and Lovo cells followed by *EPAS1* suppression was studied by colony formation assay.^33^ For this, equal numbers of SW480 and Lovo cells were seeded in six‐well plates and then SW480 and Lovo cells were transfected with *EPAS1* and scrambled siRNA. After that, treated and control cells were cultured at 37℃ in 5% CO_2_ until colonies were noted under a microscope. When the microscopic clones were detected, cells were fixed using 70% ice‐cold ethanol at room temperature for 15 min. Then, the fixed clones were stained with 0.5% crystal violet at room temperature for 2 h. The plates were then air‐dried followed by washing with tap water. Finally, images of the plates were taken and clone formation rates were calculated.

### 2.12 Wound healing assay

A scratch/wound healing assay was used to study the impact of *EPAS1* silencing of colon cancer cells migration capacity followed the protocol previously published.^34^ For this experiment, cancer cells (SW480 and Lovo) were cultured up to 70–80% confluency as a monolayer in a six‐wells plate. Then, the scratches were generated using 200 μl pipette tips through the middle of the plate. Subsequently, *EPAS1* and controls siRNAs were added to the wells and then the plates were left for recovering the generated wounds. During the experiment, images of the cells were taken to examine the changes on days 0–2. Finally, the wound areas on different days were measured and compared with Image J 1.48 software.

### 2.13 Invasion and migration assay

*EPAS1* silencing effects on cancer cell invasion and migration were examined by the basement membrane extract (BME)‐coated invasion assay kit (Trevigen Inc.) as previously described.^35^ In brief, cells (SW480 and Lovo) were grown up to 80% confluency and then cultured 24 h in serum‐free medium. These serum‐starved cells were collected and resuspended (1 × 10^6^ cells/ml) in growth medium. Then, 50 µl of cell suspension was added at the top chamber of 96‐wells plate and transfection complex (*EPAS1* or scramble siRNA, HiPerFect, a transfection reagent) was added to the cells. The cells treated with *EPAS1* and scramble siRNA were cultured for another 48 h in complete growth media. Then, cell dissociation solution/Calcein AM (100 µl) was added in the lower chamber. These Calcein AM internalised to the invaded and migrated cells, which was cleaved by intracellular esterase, resulting in the generation of a bright fluorophore. The generated fluorescence (equivalent to the invaded cells) was measured using POLARstar Omega multi‐mode microplate reader (BMGLABTECH).

### 2.14 Statistical analysis

In statistical analysis, chi‐square test, likelihood ratio and Fisher's exact test were used for comparisons between variable groups. The experiments were carried out in triplicate and Statistical Package for Social Sciences for Windows (version 27.0, IBM SPSS Inc.) was used to execute the analysis. Kaplan‐Meier method was used for survival analysis. The quantitative data are presented as mean ±SD (standard deviation) and the significance level was taken at *p* < 0.05. *p < 0.05, **p < 0.01 and ***p < 0.001 in comparison to that of control groups.

## 3 RESULTS

### 3.1 *EPAS1* DNA amplification and mRNA overexpression in colorectal cancers

**FIGURE 2:**
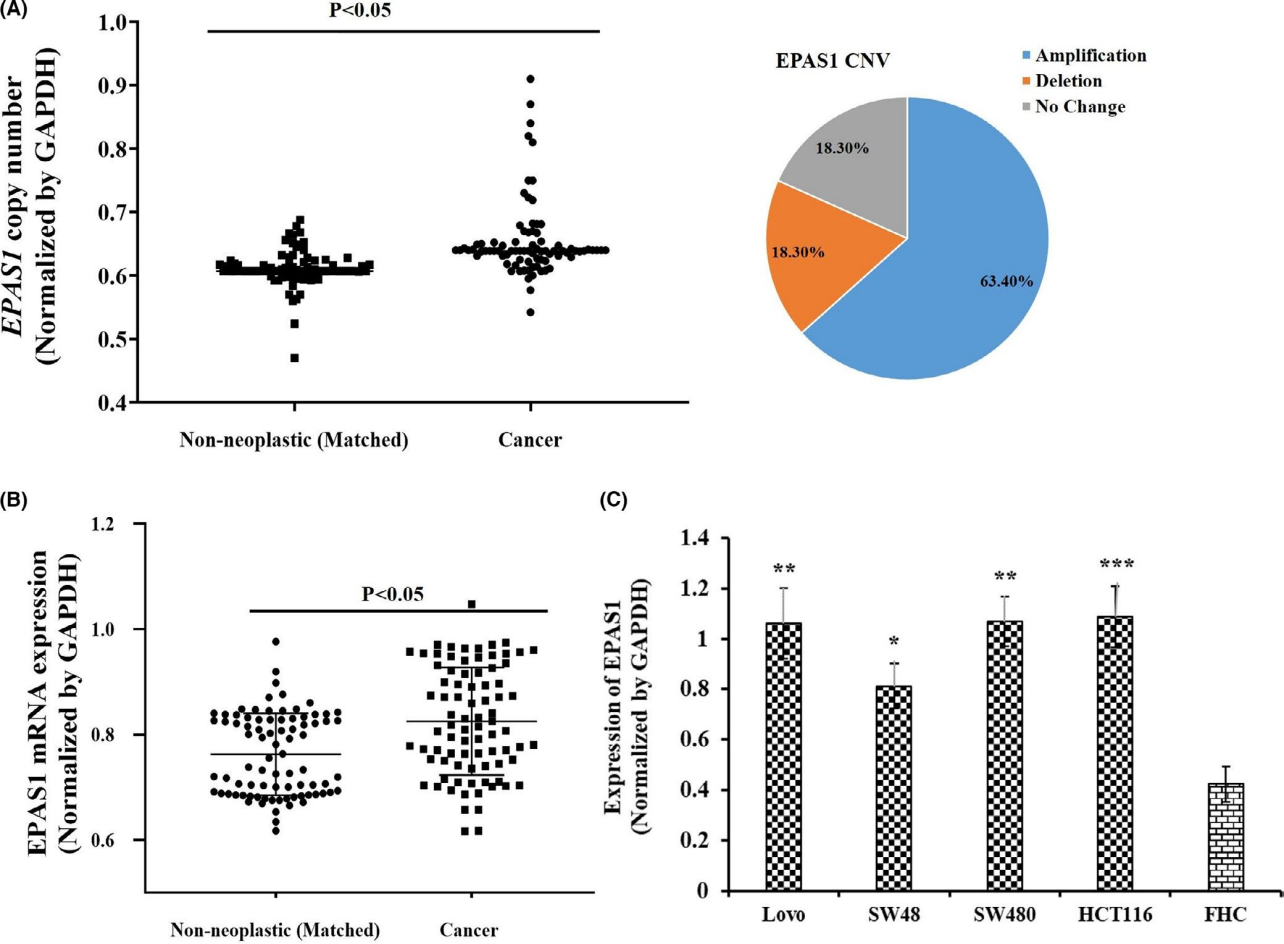
Deregulation of *EPAS1* DNA number and mRNA expression in colorectal carcinoma (CRC) and cancer cells. (A) Tissues from CRC had shown a significant amplified *EPAS1* DNA number when compared to that of matched non‐cancerous samples. (B) Likewise, CRC patients showed a significant EPAS1 overexpression when compared to that of matched non‐cancerous samples (*p *< 0.05). (C) Colon cancer cells derived from different stages exhibited higher *EPAS1* mRNA when compared with non‐cancer cells (FHC)

In CRC, 63.4% (*n* = 52/82) of cancer tissue samples showed *EPAS1* copy number amplification, whereas 18.3% (*n* = 15/82) exhibited *EPAS1* DNA deletion when compared to the matched non‐cancer tissues (Figure 2A). However, other samples (18.3%; *n* = 15/82) showed no alterations in *EPAS1* DNA numbers. The association of *EPAS1* DNA number changes with clinicopathological parameters of patients is shown in Table 1. The trends of *EPAS1* copy number amplification were related to larger tumour size, presence of tumour perforation and occurrence of synchronous CRCs (Table 1). More perceivably, 78% (*n* = 28/38) of patients having larger tumour (larger than 40 mm in diameter) showed amplified *EPAS1* DNA when compared to those of 54% (*n* = 24/44) of the patient bearing smaller tumour (40 mm or smaller in diameter) (Table 1). Similarly, CRC with perforation had a higher *EPAS1* DNA amplification when compared with those without perforation (90% vs. 60%; Table 1). Importantly, 100% (*n* = 7/7) of patients with synchronous CRC showed *EPAS1* DNA amplification, whereas only 60% (*n* = 45/75) of patients without synchronous CRC showed *EPAS1* DNA amplification (*p *= 0.03; Table 1).

*EPAS1* mRNA expression in CRCs and adjacent non‐cancerous tissues is shown in Figure 2B. Expression of *EPAS1* mRNA in cancer was significantly (*p *< 0.001) higher when compared to that in matched non‐cancer tissues (0.83 ± 0.05 vs. 0.76 ± 0.06). In addition, the expression of *EPAS1* mRNA in colon cancer cells was significantly higher when compared with non‐neoplastic colon epithelial cells (Figure 2C). Amongst the CRCs, 72% (*n* = 59/82) patients exhibited overexpressed *EPAS1* mRNA whereas only 28% (*n* = 23/82) patients had shown lower expression of *EPAS1* mRNA.

Expression of *EPAS1* mRNA was correlated with the clinicopathological factors such as T‐stage, N‐stage, presence of associated adenoma, use of pre‐operative chemo‐radiotherapy and the ethnic origin of patients with colorectal adenocarcinomas (Table 2). Overall, 81% (*n* = 35/43) of CRC having associated adenoma exhibited high *EPAS1* mRNA expression, whereas 59% (*n* = 23/39) of CRC having no associated adenoma had high *EPAS1* mRNA expression (*p *= 0.02). In addition, higher frequency (more than 75%) of cancers of T‐stages one to three exhibited high *EPAS1* mRNA expression, while only 43% of cancers of T‐stage four showed high *EPAS1* mRNA expression (*p *= 0.01). Similarly, high *EPAS1* mRNA expression was associated with CRC without lymph node metastasis (*p *= 0.03). Importantly, *EPAS1* mRNA overexpression was noted more often in cancers without pre‐operative chemo‐radiotherapy (73.7% vs. 33.3%). In addition, 81.4% (*n* = 35/43) of cancers with associated adenoma showed *EPAS1* mRNA overexpression, whereas 59% (*n* = 23/39) of cancer without associated adenoma CRC had shown *EPAS1* mRNA overexpression (*p *= 0.02; Table 2). Interestingly, 65.7% (*n* = 44/67) of cancers from patients born in Australia or New Zealand showed *EPAS1* mRNA overexpression, whereas 93.3% (*n* = 14/15) of cancers from patients born overseas exhibited *EPAS1* mRNA overexpression (*p *= 0.02; Table 2).

**TABLE 2:** Relationship between *EPAS1* mRNA expression and Clinicopathological factors of colorectal carcinoma (CRC) patients

Features	Number	High expression	Low expression	*p*‐value
Total patients	82	58 (70.7%)	24 (29.3%)	—
Gender Male	40 (48.8%)	26 (65.0%)	14 (35.0%)	0.19
Female	42 (51.2%)	32 (76.2%)	10 (23.8%)	
Age ≤70	40 (48.8%)	27 (67.5%)	13 (32.5%)	0.35
>70 Birthplace	42 (51.2%)	31 (73.8%)	11 (26.2%)	
Australia/NZ born	67 (81.7%)	44 (65.7%)	23 (34.3%)	**0.02**
Overseas born	15 (18.3%)	14 (93.3%)	1 (6.7%)	
Site				
Proximal	68 (82.9%)	51 (75.0%)	17 (25.0%)	0.06
Distal	14 (17.1%)	7 (50.0 %)	7 (50.0%)	
Size				
≤40	44 (53.7%)	32 (72.7%)	12 (27.3%)	0.42
>40	38 (46.3%)	26 (68.4%)	12 (31.6%)	
Perforation				
Yes	10 (12.2%)	5 (50.0%)	5 (50.0%)	0.12
No	72 (87.8%)	53 (73.6%)	19 (26.4%)	
Associated adenoma				
Yes	43 (52.4%)	35 (81.4%)	8 (18.6%)	**0.02**
No	39 (47.6%)	23 (59.0%)	16 (41.0%)	
Grade				
Well	12 (14.6%)	9 (75.0%)	3 (25.0%)	0.78
Moderate	57 (69.5%)	39 (68.4%)	18 (31.6%)	
Poor	13 (15.9%)	10 (76.9%)	3 (23.1%)	
T‐stage				
I	2 (2.4%)	2 (100%)	0 (0.0%)	**0.01**
II	8 (9.8%)	6 (75.0%)	2 (25.0%)	
III	51 (62.2%)	41 (80.4%)	10 (19.6%)	
IV	21 (25.6%)	9 (42.9%)	12 (57.1%)	
N‐stage				
Yes	37 (45.1%)	22 (59.5%)	15 (40.5%)	**0.03**
No	45 (54.9%)	36 (80.0%)	9 (20.0%)	
Distant metastasis				
Yes	19 (23.2%)	13 (68.4%)	6 (31.6%)	0.50
No	63 (76.8%)	45 (71.4%)	18 (28.6%)	
Pathological stage				
I & II	43 (52.4%)	34 (79.1%)	9 (20.9%)	0.06
III & IV	39 (47.6%)	24 (61.5%)	15 (38.5%)	
Pre‐operative chemo‐radiotherapy				
Yes	6 (7.3%)	2 (33.3%)	4 (66.7%)	**0.05**
No	76 (92.7%)	56 (73.7%)	20 (26.3%)	

Additionally, the overall median follow‐up of CRC patients in the current study was 59 months. It was noted that the pathological stages of cancer associated with survival rates of the patients (*p *= 0.0001). Patients with CRC having copy number amplification and expressing higher *EPAS1* mRNA had better survival time (4 and 5 months, respectively) when compared to those with *EPAS1* copy number deletion and lower mRNA expression. Nonetheless, the survival time difference between the groups did not reach a statistical significance (*p *> 0.05).

### 3.2 Detection of *EPAS1* variant in colorectal tissues

**FIGURE 3:**
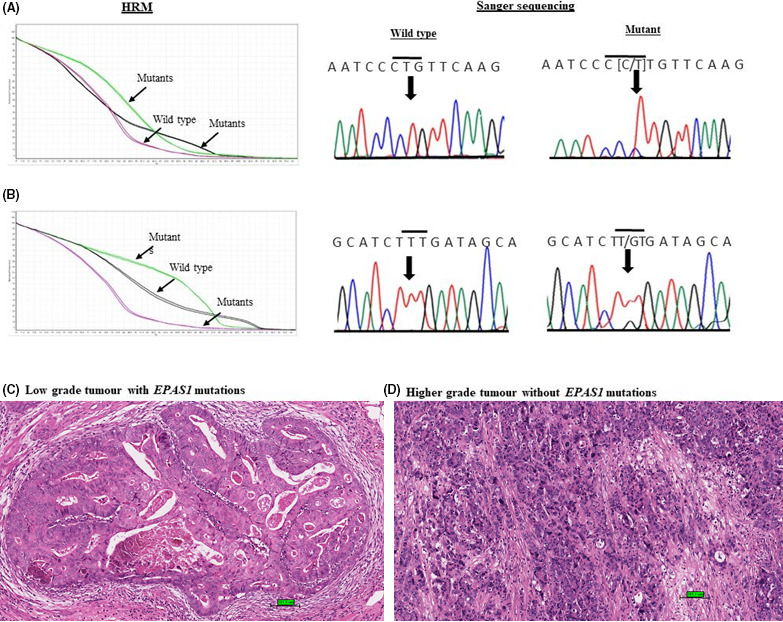
Novel mutations in *EPAS1* identifies in colorectal carcinomas (CRCs). (A) Comparative HRM curve and chromatograph for the synonymous variant c.1084C>T (p.L362L). (B) Comparative HRM curve and chromatograph for the substitutional variant c.1121T>G (p.F374C). (C) Histology of low grade (moderately differentiated) CRC harbouring *EPAS1* mutations (scale bar = 100 μm). (D) Histology of high‐grade (poorly differentiated) CRC without *EPAS1* mutations (scale bare = 100 μm)

*EPAS1* sequence variants were first suspected based on the distinctive HRM melting curve and then validated by Sanger sequencing. In colorectal cancer, 16% (*n* = 13/82) patients had shown *EPAS1* mutations in the present study (Table 3). All the mutations were detected in cancerous tissues and no mutation was found in adjacent non‐neoplastic tissues. Amongst these mutant samples, we have identified two variants (c.1084C>T and c.1121T>G) in the coding region of *EPAS1* (Figure 3A,B). Of the two mutations, c.1121T>G (p.F374C) was identified as substitutional mutations, whereas c.1084C>T (p.L362L) was a synonymous mutation.

**TABLE 3:** Identified variants and their impacts on the protein structure and function of EPAS1 in colorectal carcinoma (CRC)

Sample ID	Copy no. change	mRNA expression	Change in DNA sequence	Change in protein sequence	Effect on protein features	*In silico* prediction		
						Mutation taster	*PROVEAN*	*SIFT*
P6	Amplification	High	c.1084C>T c.1121T>G	L362L F374C	No amino acid sequence changed Changed amino acid sequence Might affect protein features Changes splice site	Diseases causing	Neutral Deleterious	Tolerated Damaging
P16	Amplification	High	c.1121T>G	F374C	Changed amino acid sequence Might affect protein features Changes splice site	Diseases causing	Deleterious	Damaging
P20	Deletion	No change	c.1121T>G	F374C	Changed amino acid sequence Might affect protein features Changes splice site	Diseases causing	Deleterious	Damaging
P21	Amplification	High	c.1084C>T	L362L	No amino acid sequence changed	Diseases causing	Neutral	Tolerated
P26	Amplification	High	c.1121T>G	F374C	Changed amino acid sequence Might affect protein features Changes splice site	Diseases causing	Deleterious	Damaging
P41	Deletion	Low	c.1121T>G	F374C	Changed amino acid sequence Might affect protein features Changes splice site	Diseases causing	Deleterious	Damaging
P57	No change	High	c.1121T>G	F374C	Changed amino acid sequence Might affect protein features Changes splice site	Diseases causing	Deleterious	Damaging
P62	Amplification	High	c.1121T>G	F374C	Changed amino acid sequence Might affect protein features Changes splice site	Diseases causing	Deleterious	Damaging
P78	Amplification	No change	c.1121T>G	F374C	Changed amino acid sequence Might affect protein features Changes splice site	Diseases causing	Deleterious	Damaging
P83	Amplification	High	c.1084C>T c.1121T>G	L362L F374C	No amino acid sequence changed Changed amino acid sequence Might affect protein features Changes splice site	Diseases causing	Neutral Deleterious	Tolerated Damaging
P103	Amplification	High	c.1121T>G	F374C	Changed amino acid sequence Might affect protein features Changes splice site	Diseases causing	Deleterious	Damaging

The probable impacts of the mutations on protein structures and functionality projected by Mutation Taster, PROVEAN and SIFT are shown in Table 3. It was noted that the effects of the variants detected in this study on the functionality of protein were predicted as deleterious or damaging (Table 3). Furthermore, to the best of our knowledge, ExAc and 1000 Genomes variant databases and PubMed search could not identify the detected variants.

The association of *EPAS1* mutations with clinicopathological parameter in patients with CRC is shown in Table 4. Mutations in the *EPAS1* sequence were statistically correlated with the absence of cancer perforation, high tumour grade and pathological stage of patients with CRC (Figure 3C,D). A 39% (*n* = 5/13) of patients with CRC bearing *EPAS1* mutations were grade 3, while 8% (*n* = 1/12) and 12% (*n *= 7/57) patients with CRC having *EPAS1* mutations were grade 1 or grade 2, respectively (*p *= 0.04). In addition, 100% (*n* = 10/10) of CRC with cancer perforation showed no mutations in *EPAS1* sequence, whereas 18% (*n* = 13/82) of CRC without mutations in *EPAS1* had cancer perforation (*p *= 0.05).

**TABLE 4:** Relationship between *EPAS1* mutations and Clinicopathological factors of colorectal carcinoma (CRC) patients

Features	Number	Negative	Positive	*p*‐value
Total patients	82	69 (84.1%)	13 (15.9%)	—
Gender Male	40 (48.8%)	36 (90.0%)	4 (10.0%)	0.13
Female	42 (51.2%)	33 (78.6%)	9 (21.4%)	
Age ≤70	40 (48.8%)	35 (87.5%)	5 (12.5%)	0.30
>70 Site	42 (51.2%)	34 (81.0%)	8 (19.0%)	
Proximal	68 (82.9%)	58 (85.3%)	10 (14.7%)	0.38
Distal	14 (17.1%)	11 (78.6 %)	3 (21.4%)	
Size				
≤40	44 (53.7%)	35 (79.5%)	9 (20.5%)	0.17
>40	38 (46.3%)	34 (89.5%)	4 (10.5%)	
Perforation				
Yes	10 (12.2%)	10 (100%)	0 (0.0%)	**0.05**
No	72 (87.8%)	59 (81.9%)	13 (18.1%)	
Synchronous cancer				
Yes	7 (8.5%)	6 (85.7%)	1 (14.3%)	0.69
No	75 (91.5%)	63 (84.0%)	12 (16.0%)	
Grade				
Well	12 (14.6%)	11 (91.7%)	1 (8.3%)	**0.04**
Moderate	57 (69.5%)	50 (87.7%)	7 (12.3%)	
Poor	13 (15.9%)	8 (61.5%)	5 (38.5%)	
Distant metastasis				
Yes	19 (23.2%)	15 (78.9%)	4 (21.1%)	0.34
No	63 (76.8%)	54 (85.7%)	9 (14.3%)	
Pathological stage				
I	9 (10.9%)	7 (77.8%)	2 (22.2%)	**0.05**
II	34 (41.5%)	27 (79.4%)	7 (20.6%)	
III	19 (23.2%)	19 (100%)	0 (0.0%)	
IV	20 (24.4%)	16 (80.0%)	4 (20.0%)	
Chemo‐radiotherapy				
Yes	6 (7.3%)	5 (83.3%)	1 (16.7%)	0.65
No	76 (92.7%)	64 (84.2%)	12 (15.8%)	

Note

Synchronous cancer means more than one primary cancer in the colon.

### 3.3 Relationship of *EPAS1* copy number change, level of mRNA expression and mutations in crc patients

**FIGURE 4:**
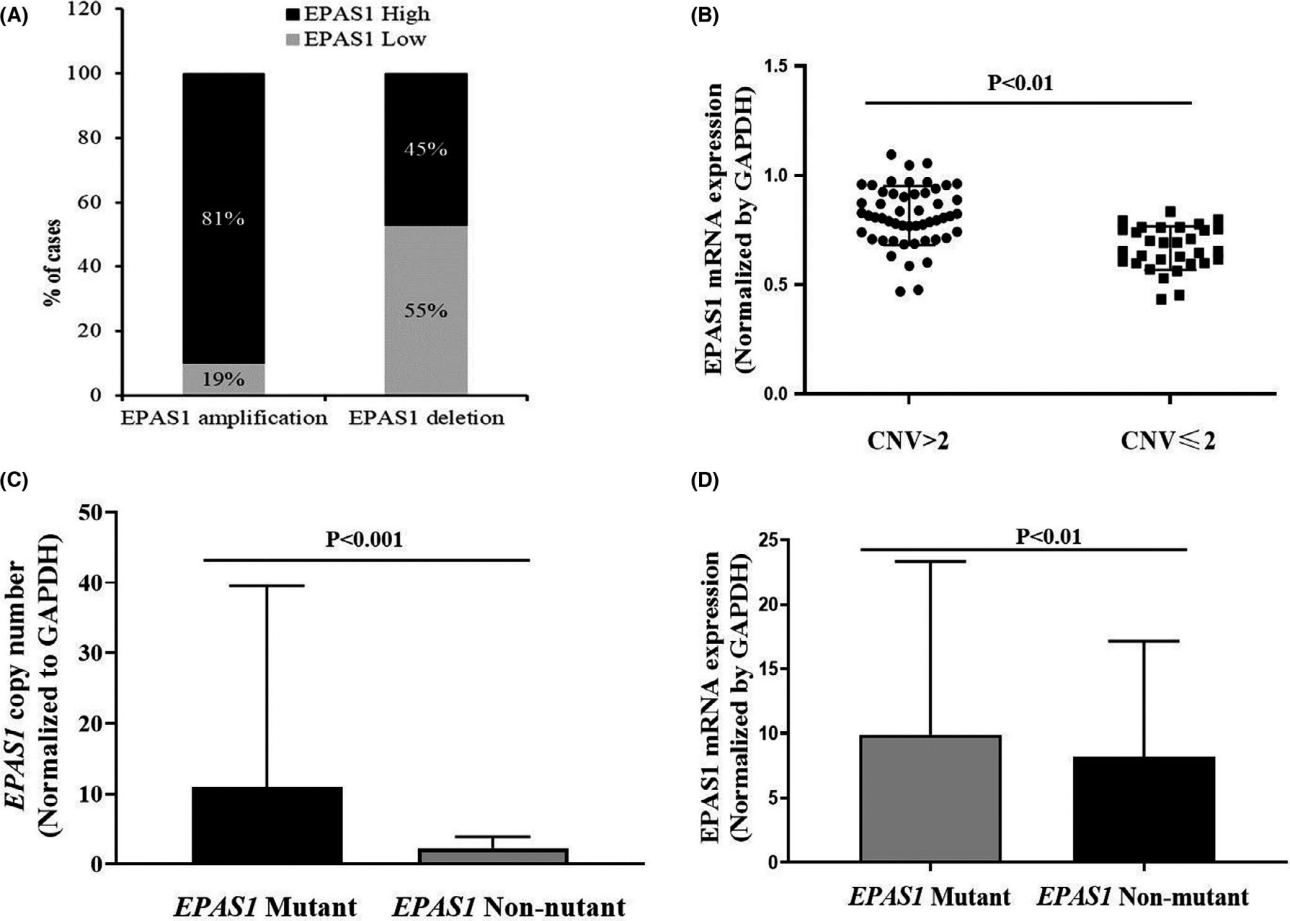
Correlation amongst *EPAS1* copy number variation, expression of mRNA and mutations. (A) *EPAS1* DNA number variation and mRNA expression relationship. Amplified *EPAS1* copy number associated with higher mRNA expression significantly (*p *< 0.0001). (B) *EPAS1* mRNA distribution in colorectal carcinoma (CRC) patients categorised based on *EPAS1* DNA number ≤2 and >2. Patients with CRC having higher DNA copy number showed overexpression of *EPAS1* in mRNA level. (C) Patients with CRC harbouring *EPAS1* mutations exhibited amplified DNA number when compared to that of mutation‐negative tissues. (D) Also, patients having *EPAS1* mutations had shown a significant mRNA overexpression (mRNA) in comparison to mutation‐negative tissues

A positive correlation (*r *= 0.476; *p *= 0.01, Fisher exact test) between *EPAS1* copy number amplification and mRNA overexpression was noted in the present study. Ninety percent (*n* = 47/52) of CRC patients having amplified *EPAS1* copy number exhibited higher *EPAS1* mRNA level, whereas lower *EPAS1* mRNA expression was only found in 53% (*n* = 16/30) CRC patients with lower *EPAS1* copy number (Figure 4A). Similarly, the expression of *EPAS1* mRNA changed significantly along the alterations of DNA number changes (Figure 4B). The expression of *EPAS1* mRNA showed a significant difference when CRC having *EPAS1* copy number variation of more than 2 (CNV>2) is compared with copy number variation equal or <2 (CNV≤2). CRCs harbouring *EPAS1* mutations exhibited substantial amplified and higher DNA number variations when compared to those of CRC without the mutations (Figure 4C). Similarly, mutation‐positive CRC tissues had shown notable *EPAS1* mRNA overexpression in comparison to those of non‐mutated cancer samples (Figure 4D).

### 3.4 *EPAS1* silencing induced reduced proliferation and colony formation of colon cancer cells

**FIGURE 5:**
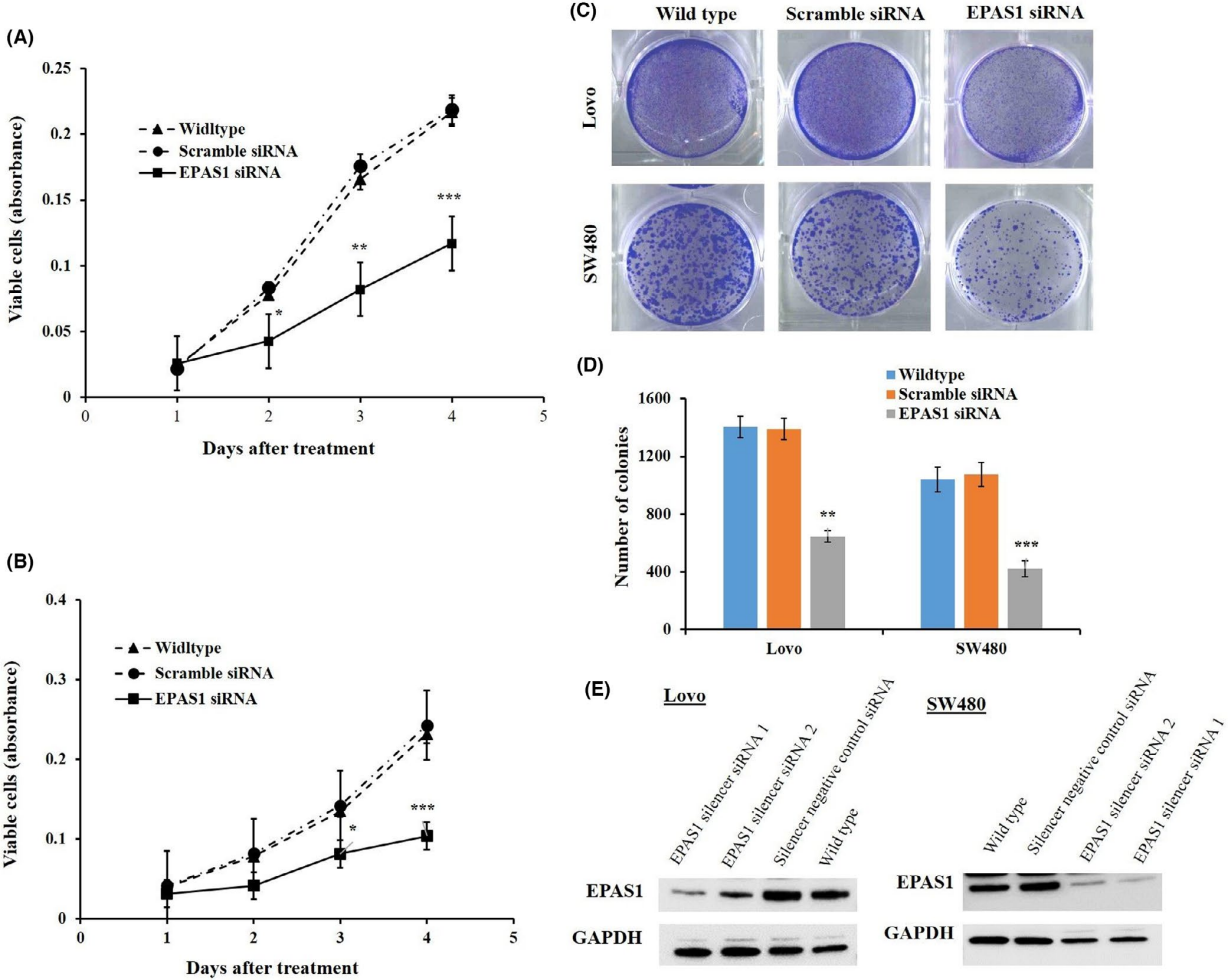
Silencing of *EPAS1* inhibited colon cancer cell growth and proliferation. Silencing of *EPAS1* with siRNA silencer sequence in colon cancer SW480 (A) and Lovo (B), cells induced significant inhibition of cell proliferation at various time points in vitro in comparison to that of control cells. Also, *EPAS1* suppression caused inhibition of colony formation capacity of SW480 (C) and Lovo (D) cells when compared to that of control cells. (E) EPAS1 detection in colon cancer cells treated with *EPAS1* and scramble siRNA. siRNA treatment notably silences EPAS1 protein expression. Two siRNA specific for *EPAS1* (EPAS1 silencer siRNA 1 and EPAS1 silencer siRNA 2) were used to the knockdown EPAS1 expression whereas Silencer negative control siRNA was as the control (EPAS1 positive) group

*EPAS1* was silenced in colon cancer cells by siRNA followed by cell proliferation, invasion and migration of *EPAS1* suppressed (SW480^−^ *^EPAS1^*, Lovo^−^ *^EPAS1^*) and controlled (SW480^+Scr^ and Lovo^+Scr^, SW480^wildtype^ and Lovo^wildtype^) cells were examined at a different time interval (days 0–3). SW480^−^ *^EPAS1^* and Lovo^−^ *^EPAS1^* cells exhibited a remarkably reduced proliferation of cells in comparison to that of controls (SW480^+Scr^, Lovo^+Scr^, SW480^wildtype^ and Lovo^wildtype^; Figure 5). SW480^−^ *^EPAS1^* cells showed 45%, 50% and 46% reduced cell proliferation when compared to that of SW480^+Scr^ cells on day 1, day 2 and day 3, respectively (Figure 5A). Similarly, Lovo^−^ *^EPAS1^* cells exhibited 40%, 47% and 55% reduced proliferation in comparison to that of Lovo^+Scr^ cells on day 1, day 2 and day 3, respectively (Figure 5B).

Suppression of *EPAS1* in colon cancer cells (SW480^−^ *^EPAS1^* and Lovo^−^ *^EPAS1^*) induced the reduction of colony formation rates significantly when compared to SW480^+Scr^ and Lovo^+Scr^ and non‐transfected SW480^wildtype^ & Lovo^wildtype^ control colon cancer cells (Figure 5C,D). It was noted that SW480^−^ *^EPAS1^* cells had shown a 53% reduction in colony formation capacity in comparison to SW480^+Scr^ cells, whereas Lovo^−^ *^EPAS1^* cells exhibited a 61% reduction in colony formation when compared to that of Lovo^+Scr^ cells (Figure 5D).

### 3.5 *EPAS1* suppression inhibited wound healing, migration and invasion of colon cancer cells

**FIGURE 6:**
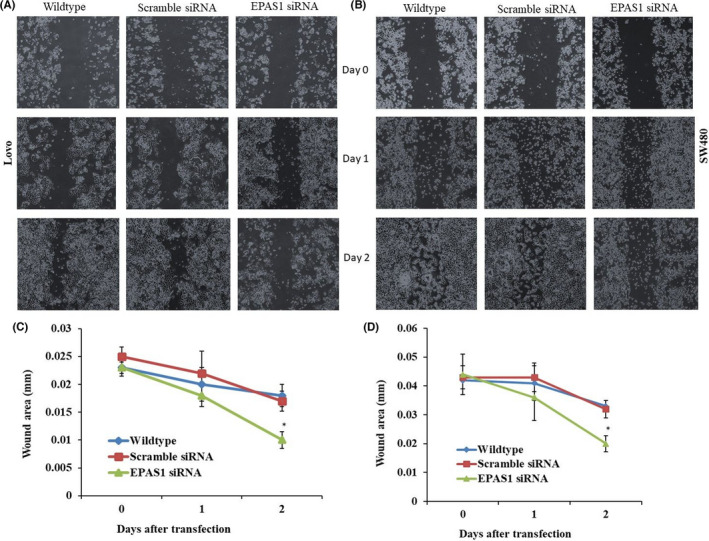
*EPAS1* suppression induced the inhibition of the wound healing capacity of colon cancer cells. *EPAS1* silencing caused the inhibition of migration property capacity of colon cancer cells, resulting in slow healing of the wounds when compared to controls (A, B). Wounds areas were recorded for all the cells group on different time points in three independent measurements (C, D)

SW480^−^ *^EPAS1^* and Lovo^−^ *^EPAS1^* (*EPAS1* silenced) colon cancer cells exhibited significant inhibition of wound healing, barrier penetration and migration when compared to that of controls (SW480^+Scr^ & Lovo^+Scr^) and non‐transfected wild‐type (SW480^wildtype^ & Lovo^wildtype^) cells. SW480^−^ *^EPAS1^* & Lovo^−^ *^EPAS1^* cells exhibited reduced migration than SW480^+Scr^ & Lovo^+Scr^ and SW480^wildtype^ & Lovo^wildtype^ cells (Figure 6). SW480^−^ *^EPAS1^* & Lovo^−^ *^EPAS1^* cells cured the wounds slowly and took a significantly longer time to heal the wounds (Figure 6).

**FIGURE 7:**
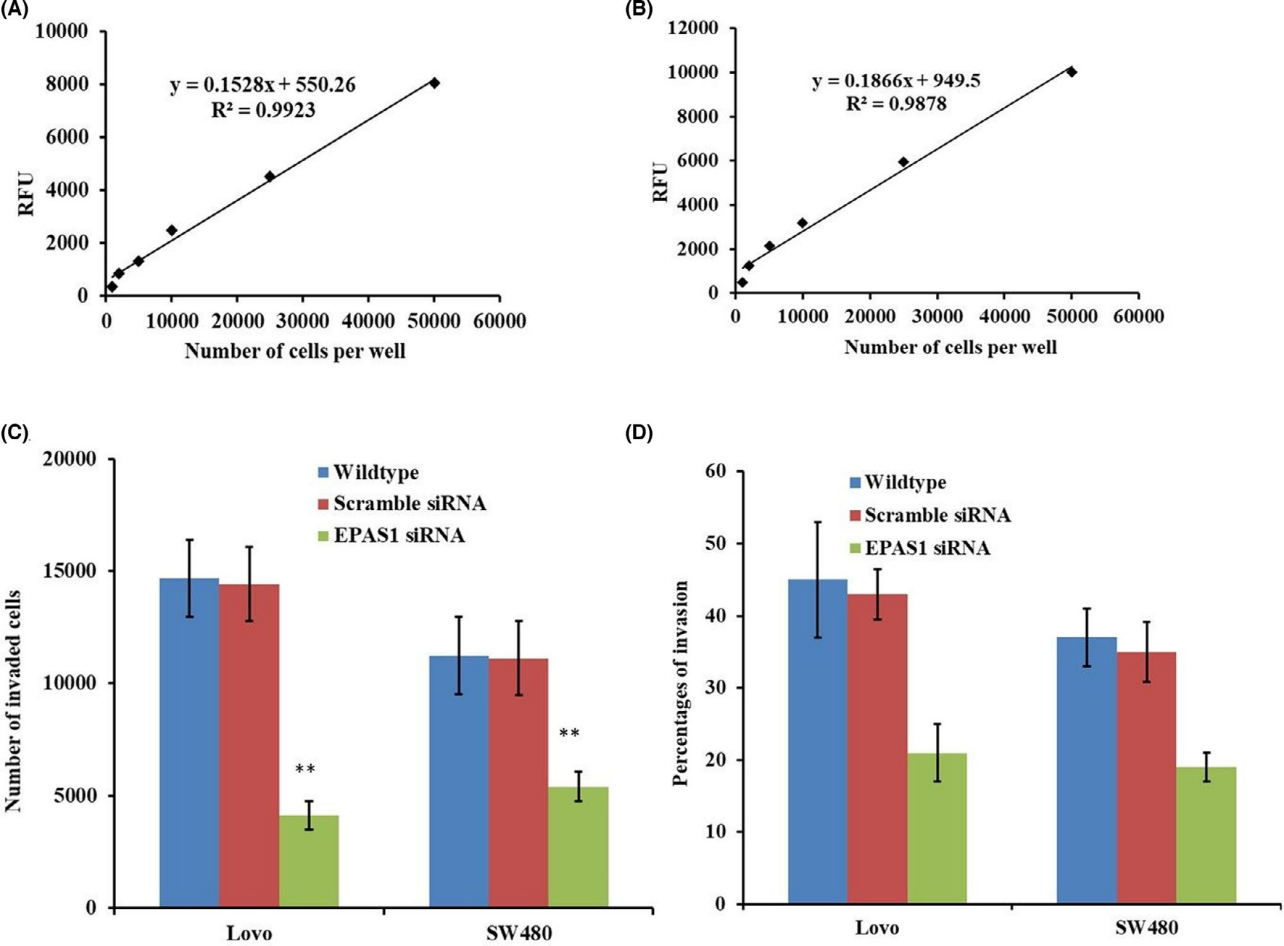
Inhibition of *EPAS1* induced the inhibition of invasion and migration of colon cancer cells. SW480 and Lovo colon cancer cells were harvested, incubated for 1 h with Calcein AM and assayed for fluorescence. Then the standard curve for SW480 (A) and Lovo (B) cells were generated. Significantly reduced population of cells had shown barrier penetration and migration in *EPAS1* suppressed cells group in comparison to control groups (C, D)

Also, SW480^−^ *^EPAS1^* & Lovo^−^ *^EPAS1^* cells showed lower invasion and migration capacity BME‐coated invasion chamber in comparison to that of SW480^+Scr^ & Lovo^+Scr^ and SW480^wildtype^ & Lovo^wildtype^ control cells (Figure 7). The invaded SW480^−^ *^EPAS1^* and Lovo^−^ *^EPAS1^* cells were low in number when compared to that of SW480^+Scr^ & Lovo^+Scr^ and SW480^wildtype^ & Lovo^wildtype^ cells (Figure 7).

## 4 DISCUSSION

The present study reported insights into the functional roles of *EPAS1* in CRC and evaluated its clinical significance by studying copy number changes, mRNA expression and mutations screening in patients with CRC. Our results indicate that *EPAS1* has important roles in the pathogenesis of CRC via modulating cellular proliferation, invasion and migration, by acting as a tumour promoter gene.

In cancer, copy number amplification and overexpression of oncogenes are being frequently uses as the disease progression biomarkers.^36^ Amplification of DNA copy number (63.4%; *n *= 52/82) followed by mRNA overexpression (72%; *n* = 59/82) of *EPAS1* tissues implies its cancer‐promoting properties in patients with CRC. Previous studies also noted the overexpression of *EPAS1* both in mRNA and protein levels in CRC.^17^, ^18^ However, some other studies noted a downregulation of *EPAS1* expression in CRC.^13^, ^15^ Thus, the expression pattern and its molecular roles in CRC are inconsistent.

In the present study, a trend of association of *EPAS1* DNA amplification with larger tumour, the presence of perforation and higher incidence synchronous tumours are in keeping with cancer‐promoting function of *EPAS1* in CRC. In addition, the relationship of *EPAS1* mRNA overexpression with less advanced pathological stages CRCs indicates its role in the initiation of the pathogenesis of CRC. However, a small number of cases from patients with early cancer stages is a limitation in the current study. Overexpression of *EPAS1* mRNA in CRC of patients without pre‐operative chemo‐radiotherapy and reduced expression in CRC of patients receiving pre‐operative chemo‐radiotherapy further confirms the oncogenic potential of *EPAS1* in CRC. Furthermore, the association of *EPAS1* mRNA overexpression with associated adenoma indicates its roles in promoting the formation of multiple tumours in patients with CRC (Table 4). Therefore, the expression of *EPAS1* mRNA in patients with CRC has the potential of prognostic significance.

Aberrations of DNA copy numbers is a common change in cancer cells, which can lead to altered gene expressions, thus, can play a key role in the initiation and development of CRC.^37^ The Cancer Genome Atlas (TCGA) data analysis of EPAS1 DNA copy number changes showed that 24% of patients had amplified *EPAS1* DNA number, whereas 40% of patients had shown deleted *EPAS1* DNA number (https://portal.gdc.cancer.gov/). The statistical relationship between *amplified* DNA number and higher mRNA expression of *EPAS1* in patients with CRC in this study implied that the hypoxic tumour niches caused molecular changes in *EPAS1*. These alterations can promote carcinogenesis in colorectum. In addition, higher DNA number and increased mRNA expression level in patients with CRC harbouring *EPAS1* mutations indicated the concerted molecular deregulation of *EPAS1* in CRC. However, a previous study reported that the complete deletion of *EPAS1* associated with Lynch syndrome, a hereditary risk factors for development of CRC.^22^ Therefore, it is important to explore the different functional roles of the variants identified in the current study in the pathogenesis of CRC.

In this study, we first report for the first‐time mutations of *EPAS1* CRC patients along with their clinicopathological associations. Relationship between *EPAS1* mutations and higher tumour grade indicates that mutations in the *EPAS1* sequence contributed to the biological aggressiveness in CRC. International cancer genome consortium data mining indicates a number of mutations of *EPAS1* sequence in the number of human cancers including CRC (https://dcc.icgc.org/). The frequencies of *EPAS1* mutations in CRC patients in non‐Western populations and in population from the United States are 7.48% and 2.48%, respectively. Amongst the mutations detected, 65% (*n* = 34/54) of the mutations affect the donors. In this study, *EPAS1* mutations were detected in 16% (*n* = 13/82) CRC patients from Australia. Similarly, in this study, a significant *EPAS1* mRNA overexpression was noted in patients with CRC born overseas in comparison to those born in Australia or New Zealand. Thus, the frequency of the mutation or expression profiles of *EPAS1* in patients with CRC could differ in different ethnic groups. In silico analysis predicts that all detected variants in this study are novel and could alter the structural and functional features of the protein. The variant (c.1121T>G; F374C) could induce alterations of the primary structure of the protein, which in turn may lead to non‐functional/over functional protein. However, further studies with the functional implication of this variant are imperative to validate its actual role in the protein product. Previous studies identified a number of variants (e.g., c.1592C>T; c.1121T>A; c. 1104F>A; c.1234T>A; c.1595A>G; c.1589C>A; c.1588G>A; c.1591C>T; c.1599_1604del; c.1600_1608del; c.1615G>T) in *EPAS1* sequence in non‐familial pheochromocytomas, paragangliomas and pancreatic carcinomas.^10^, ^21^, ^38^ However, the functional roles of these variants in cancer pathogenesis are not reported in the literature. Thus, further functional investigations are needed to confirm the roles of the variants detected herein.

Roles of *EPAS1* in cancer pathogenesis have been reported in several malignancies such as pancreatic, breast and clear cell renal cell carcinomas.^6^, ^39^, ^40^ For example, siRNA mediated silencing of *EPAS1* induced reduce proliferation of cells, higher number of apoptosis and produced smaller tumour in pancreatic carcinoma xenotransplanted mouse model.^39^ Furthermore, significant regression of primary and metastatic clear cell renal cell carcinoma was noted mouse followed by the inhibition of EPAS1 with a small molecular target (PT2399).^6^ Similarly, herein, the suppression of *EPAS1* via silencer siRNA‐sequence caused the inhibition of proliferation, colony formation of colon cancer cells significantly when compared to that of control groups. These results imply that EPAS1 has the potential to be a target for therapy development for better management of patients with cancer.

Additionally, EPAS1 interacts with both VEGF and its receptor Fms‐related tyrosine kinase 1 (Flt1), thereby promotes angiogenesis.^41^ This interaction leads to VEGF and Flt1 overexpression in endothelial cells, which stimulates mature angiogenesis in wound healing in a mouse model.^29^ In addition, shRNA‐mediated silencing of *EPAS1* inhibited the cellular response and halt angiogenesis in breast cancer significantly.^40^ In this study, we have noted a similar trend where silencing of *EPAS1* in colon cancer cells remarkably suppressed migration and wound healing potential of colon cancer cells. Also, suppression of *EPAS1* in colon cancer cells caused inhibition of membrane penetrations and invasion, implying poorer metastatic ability of these cells. Therefore, therapeutic strategies silencing *EPAS1* could act as a management strategy for controlling cancer cell growth and metastasis in patients with CRC.

In conclusion, we for the first time reported novel variants of *EPAS1* sequence in patients with CRC. Aberrant expression and/or structural and functional alterations in the gene or gene product could be attributed by these mutations, which may contribute to CRC pathogenesis. Also, relationship amongst DNA number variations, mRNA expression and presence of *EPAS1* mutations in CRC with clinicopathological factors implies the prognostic significance of *EPAS1* in disease progression. Studies on genetic to clinical, functional to molecular aspects have given important information regarding *EPAS1*‐mediated tumour endorsement in CRC pathogenesis. Thus, results from this study would further improve the existing information of *EPAS1* directed molecular pathogenesis in CRC and thereby provide strategies to develop novel therapeutics for patients with CRCs.

## CONFLICT OF INTEREST

None.

## ETHICAL APPROVAL

Ethical approval for this work has been obtained from the Griffith University Human Research Ethics Committee (GU Ref No: MSC/17/10/HREC). Duly signed the copy for the consent of participation were collected from the patients used in the study.

## Data Availability

The data that support the findings of this study are available on request from the corresponding author. The data are not publicly available due to privacy or ethical restrictions.
